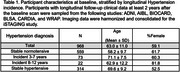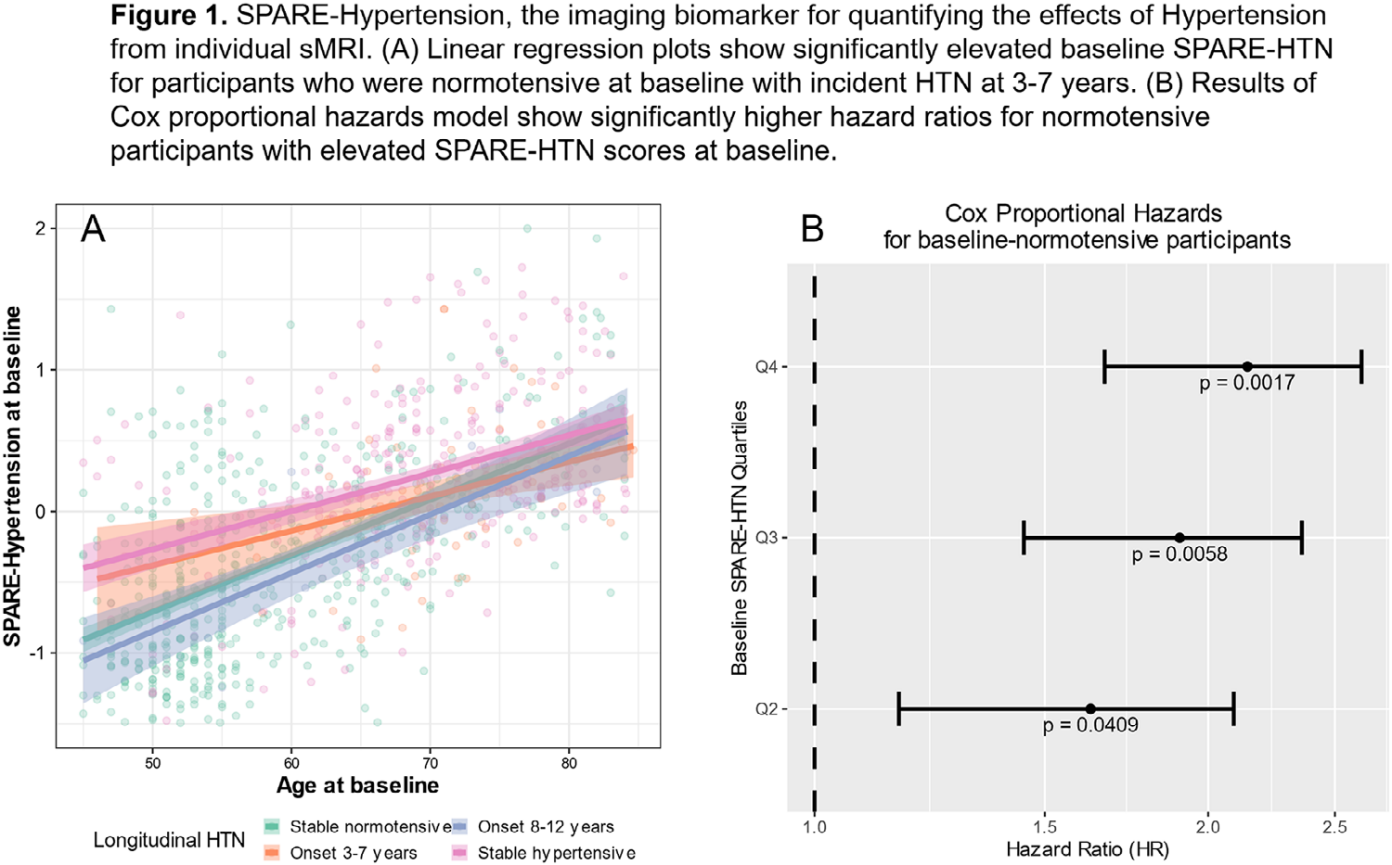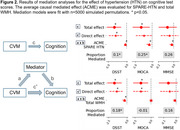# A Machine Learning‐Based MRI Marker Predicts Incident Hypertension and Mediates the Relationship Between Hypertension and Cognition

**DOI:** 10.1002/alz70862_109807

**Published:** 2025-12-23

**Authors:** Sindhuja Tirumalai Govindarajan, Elizabeth Mamourian, Dhivya Srinivasan, Guray Erus, Randa Melhem, R Nick Bryan, Haochang Shou, Ilya M. Nasrallah, Christos Davatzikos

**Affiliations:** ^1^ Artificial Intelligence in Biomedical Imaging Laboratory (AIBIL), Center for and Data Science for Integrated Diagnostics (AI2D), Perelman School of Medicine, University of Pennsylvania, Philadelphia, PA USA; ^2^ University of Pennsylvania, Philadelphia, PA USA; ^3^ Artificial Intelligence in Biomedical Imaging Laboratory, Perelman School of Medicine, University of Pennsylvania, Philadelphia, PA USA; ^4^ University of Pennsylvania Health System, Philadeplphia, PA USA; ^5^ Department of Biostatistics, Epidemiology, & Informatics, University of Pennsylvania, Philadelphia, PA USA; ^6^ Department of Radiology, University of Pennsylvania, Philadelphia, PA USA

## Abstract

**Background:**

Hypertension (HTN) is an established risk factor for neurodegeneration and dementia, supported by epidemiological and neuroimaging studies, although there is large variation in individual outcomes. We developed a machine learning (ML)‐based model, termed SPARE‐HTN, to quantify the spatial pattern of HTN‐related neurodegeneration observable in individual structural magnetic resonance images (sMRI). SPARE‐HTN demonstrates superior sensitivity compared to the most widely‐used measure of HTN‐related brain changes, correlates with cognitive performance, and detects early changes in mid‐life years. This study investigated the predictive capacity of SPARE‐HTN for incident HTN and its mediating role in the relationship between HTN and cognition.

**Methods:**

SPARE‐HTN, derived from *N* = 37,098 cognitively unimpaired individuals from diverse cohorts, was evaluated in *N* = 968 (59% female, mean age 63.0 ± 11 years) individuals with longitudinal clinical data from six studies (Table 1). Baseline SPARE‐HTN values were compared across participants categorized by longitudinal HTN status: persistently normotensive, persistently hypertensive, or incident HTN. The risk of incident HTN was evaluated among baseline normotensive participants using Cox regression model across the baseline SPARE‐HTN quartiles, adjusted for age and sex. The average causal mediated effect (ACME) of SPARE‐HTN and total white matter hyperintensity (WMH) volume on the relationship between hypertension and cognitive test scores were assessed using mediation models with 5000 simulated permutations.

**Results:**

Table 1 presents the participant characteristics at baseline, stratified by longitudinal HTN status. SPARE‐HTN was significantly elevated (Figure 1A) in participants who were normotensive at baseline but developed HTN within 3‐7 years (+0.44, *p* = 0.01), but not in those who developed HTN in 8‐12 years (*p* = 0.65). Normotensive participants with elevated baseline SPARE‐HTN scores exhibited significantly higher Cox proportional hazard ratios for HTN incidence (Figure 1B). Mediation analysis demonstrated that SPARE‐HTN mediated up to 26% of the effect of HTN on cognitive measures, whilst the conventional WMH volumes showed little mediation effect (Figure 2).

**Conclusion:**

Our ML‐based sMRI marker predicted incident HTN prior to formal clinical diagnosis, suggesting the presence of subclinical cerebrovascular changes possibly associated with blood pressure variations. These markers, particularly relevant in midlife, offer potential for informing dementia prevention trials by enabling individualized risk stratification and potentially more sensitive measurement of therapeutic efficacy.